# Compensatory gene expression potentially rescues impaired brain development in *Kit* mutant mice

**DOI:** 10.1038/s41598-023-30032-0

**Published:** 2023-03-13

**Authors:** Ryuhei Minei, Hitomi Aoki, Atsushi Ogura, Takahiro Kunisada

**Affiliations:** 1 Department of Bio-Science, Nagahama Institute of Bio-Science and Technology, Shiga, Japan; 2 Department of Tissue and Organ Development, Regeneration, and Advanced Medical Science, Graduate School of Medicine, Gifu University, 1-1 Yanagido, Gifu, 501-1194 Japan

**Keywords:** Developmental biology, Genetics, Molecular biology

## Abstract

While loss-of-function mutations in the murine dominant white *spotting/Kit (W)* locus affect a diverse array of cell lineages and organs, the brain, organ with the highest expression show the least number of defective phenotypes. We performed transcriptome analysis of the brains of *Kit*^*W*^ embryos and found prominent gene expression changes specifically in the E12.5 *Kit*^*W/W*^ homozygous mutant. Although other potentially effective changes in gene expression were observed, uniform downregulation of ribosomal protein genes and oxidative phosphorylation pathway genes specifically observed in the E12.5 brain may comprise a genetic compensation system exerting protective metabolic effects against the deleterious effect of *Kit*^*W/W*^ mutation in the developing brain.

## Introduction

The fact that 1341 of 1751 tested mouse genes showed no deleterious phenotypes after the knockout of both alleles reminded us of the possible prevailing genetic compensatory system among the individual genes^1^. Molecular mechanisms of genetic compensation have been specifically analyzed in the case X chromosome inactivation^2^; however, genetic compensation of autosomal genes leading to the lack of phenotypes in many engineered mutants has long led to various arguments^3–8^, but mechanistically has not been sufficiently explained in each case, except for some genes compensated by functionally related genes through a highly specific molecular mechanism^9,10^.

Loss-of-function mutations in the murine dominant *white spotting(W)* locus encoding the *Kit* receptor tyrosine kinase affect various cell lineages and is manifested as severe anemia, defective pigmentation, sterility, etc.^11,12^. We recently investigated the major roles of Kit receptor tyrosine kinase in early brain development, in which conditional haploinsufficiency of *Kit* induced by the neural cell-specific *Sox1-Cre* system abolished proliferation of neural stem cells and led to a hypoplastic and mostly lethal embryonic brain phenotype^13^. While no major developmental defects except some specific types of neurons^14,15^ have been reported in any type of germ line *Kit* mutant mice, and the role of Kit in developing brain tissues where it is abundantly expressed has not been determined^16–18^. Seemingly, *Kit* is nonfunctional in the developing wild-type brain despite its high expression levels. Our findings also excluded the simple assumption that *Kit* loss-of-function mutation in the brain is compensated only via a protein feedback system including the *Kit* pathway. If this was the case, harmful phenotypes of *Kit* loss-of-function mutations could have never manifested in the developing brain.

In germ-line *Kit* loss-of-function mutants, the indispensable *Kit* function could be genetically compensated during embryogenesis, whereas conditional haploinsufficiency suddenly evoked in the neural precursor cells by the *Sox1-Cre* apparatus was not fully restored by the privileged compensatory system^13^. This raises the question of what gene(s) are compensatory in the germ line *Kit* mutant brain? As the assumed compensatory system is not restricted to the transcriptional alterations of functionally related genes but is attained by a wider gene network, the first view of the compensatory system is likely to be gained by the comparative gene expression between germ line *Kit* mutant and wild-type brain tissues.

## Results

### Testing compensatory gene expression in germline *Kit* mutant brains to explain impaired brain development previously observed in the induced brain-specific haploinsufficiency of *Kit*

Prevailing *Kit* receptor tyrosine kinase expression was observed in the developing brain^16–18^; however, none of the *Kit* mutants showed phenotypic abnormalities other than either subtle impairments of higher brain functions^19^ or relatively minor perturbations to brain structure and biochemistry, including axon guidance of commissural neurons^14^ and the transduction properties of sensory neurons^15^. This could be the result of compensatory gene expression^20,21^, or just an imagined *Kit* is only expressed, but not really functioning. A recent experiment that induced haploinsufficiency of *Kit* in the early phase of brain development caused serious hypoplasia of the central nervous system (Aoki et al.^13^ and Supplemental Fig. 1a), strongly indicating that *Kit* functions in the developing brain. Therefore, we hypothesized that in well-characterized germline *Kit* mutants, some compensatory gene are expressed to substitute for Kit’s vital function at early stages of brain development. In stage-specific conditional knockout of *Kit* in the brain, the sudden decrease in Kit receptor expression and function in brain cells is supposed not to allow enough time for the assumed compensatory gene(s) to reactively increase its expression^13^. In other words, very little phenotypic disorder in the germline *Kit* mutant brain could be explained by a negative feedback system that enables seamless compensatory gene expression. For this purpose, we quantified gene expression in germline *Kit* mutant brains at various developmental stages. Since 60% of brain cells are expected to express Kit on their surface (Aoki et al.^13^; Supplemental Fig. 1b), possible gene expression changes expected to occur in those *Kit* expressing brain cells are likely to be detected by transcriptome analysis of whole brain mRNA samples.

**Figure 1:**
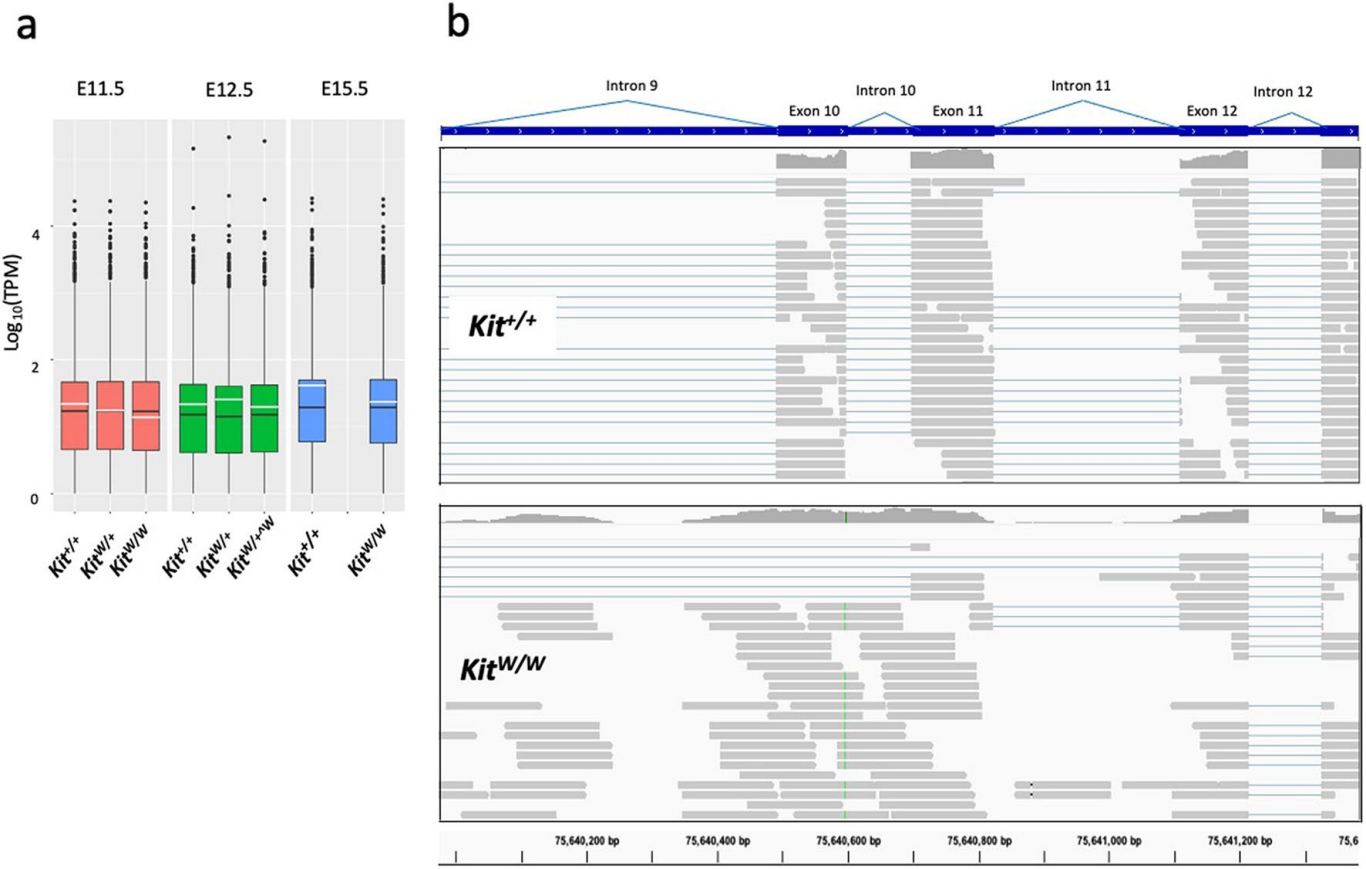
*Kit* gene expression profiles after transcriptomic analysis of *Kit*^*W*^ mutant embryonic brains. (**a**). Box plot of the analyzed genes (with the median value and 25th and 75th percentils) by RNA-seq. Black bar in each box shows the mean of all the analyzed genes and white bar is the mean of the *Kit* mRNA. TPM: Transcript per million. (**b**) Sequence alignment of each read (gray crossbar) of the Kit mRNA obtained from *Kit*^*W/W*^ mutant and *Kit*^+*/*+^ wildtype control from E12.5 embryonic brains. Histograms of the reads are shown on the top of each genotype. Thick and thin blue lines represent exons and introns of the Kit gene, respectively.

We purified mRNA from the E11.5, E12.5, and E15.5 embryonic brains of *Kit*^*W*^ germline *Kit* mutant and wild-type control mice and subjected them to RNA-seq analysis. Principal component analysis (PCA) showed that mRNA samples of each developmental stage had distinct transcriptomic profiles, indicating correct staging and purification of each sample (Supplemental Fig. 2). As expected, considerable expression of *Kit* mRNA (Fig. 1a) was observed in the brains from each developmental stage, indicating a sufficient number of cells expressing *Kit* mRNA in each brain sample. *Kit*^*W*^ with a point mutation in the 5’-splice donor site of intron 10 is known to be subjected to defective splicing of intron10 to produce non-functional *Kit* protein^22^. Sequence read histograms of *Kit* mRNA around exons 9 to 11 clearly reflected this situation: in the wild-type control brain, the bars appeared only in exon 9 and exon 10, and not in intron 10, indicating the correct splicing out of intron 10 (Fig. 1b). In contrast, bars appeared continuously between exon 10 and exon11 in each *Kit*^*W/W*^ brain (Fig. 1b), suggesting the complete failure of intron10 splicing out. These histograms of *Kit* mRNA sequences were also used to verify the genotypes of the *Kit* mutant and wild-type embryos initially determined by RT-PCR^13^. As shown in Supplemental Fig. 3, genotypes determined by RT-PCR matched those determined by the sequencing data of the *Kit* gene.

### Genome-wide comparison of gene expression in the developing *Kit*^*W/W*^ brain revealed extensive developmental stage-restricted changes in gene expression

**Figure 2:**
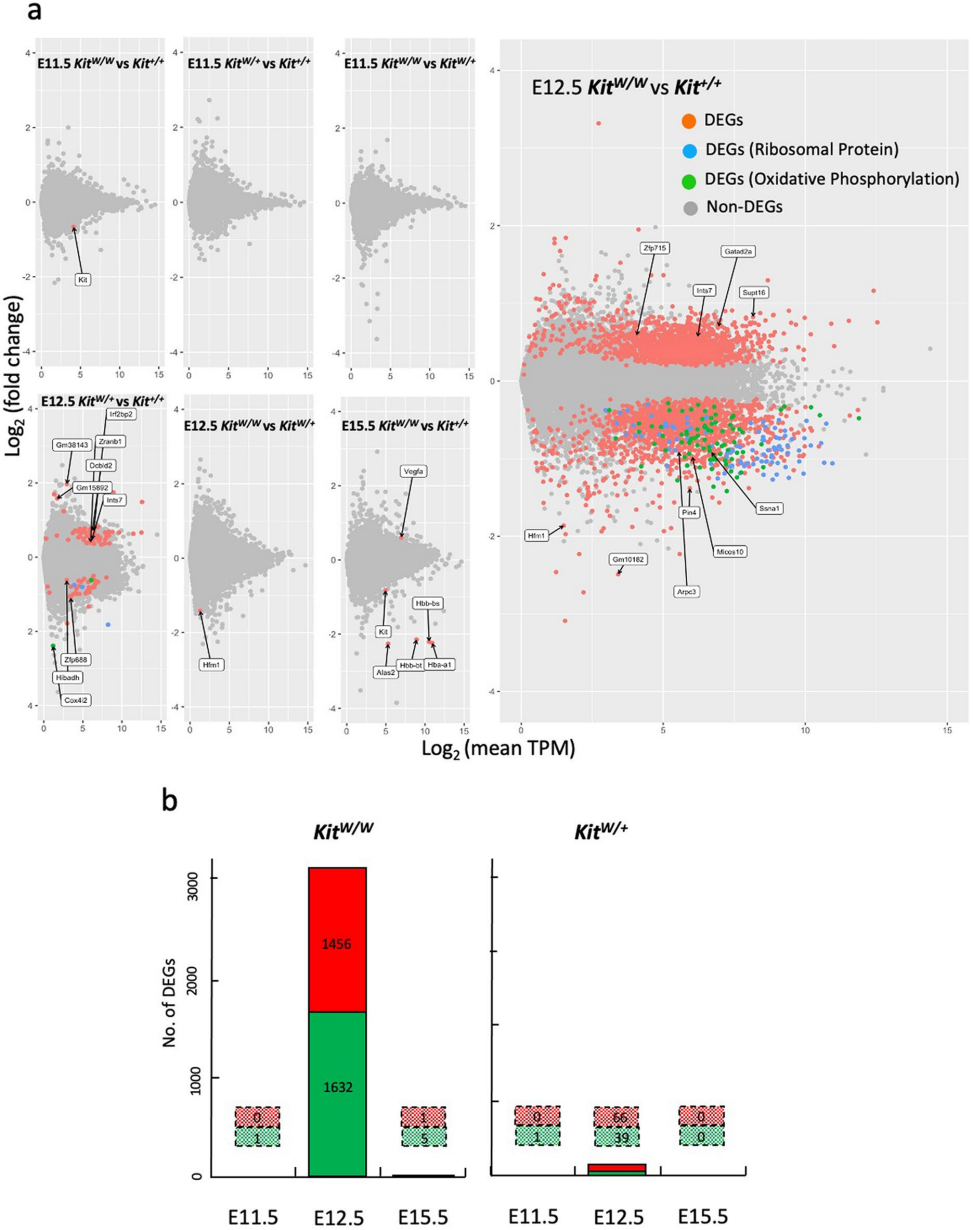
Transcriptomic analysis of developing brain reveals specific changes in gene expression in E12.5 *Kit*^*W/W*^ brain. (**a**). MA plot of the significantly differentially expressed genes (DEGs) of *Kit*^*W/W*^ and *Kit*^*W/*+^ brains relative to *Kit*^+*/*+^ brains (*p* < 0.01, ANOVA one way test) in E 11.5, E12.5 and E15.5 embryos (**b**). Number of DEGs in *Kit*^*W/W*^ and *Kit*^*W/*+^ brains compared to *Kit*^+*/*+^ brains (FDR < 0.05, Benjamini-Hochberg, two-sided) in E 11.5, E12.5 and E15.5 embryos.

Differential gene expression analysis was performed in *Kit*^*W/W*^, *Kit*^*W/*+^, and *Kit*^+*/*+^ embryonic brains using RNA-seq analysis (Fig. 2a). In the E11.5 and E15.5 brains, the gene expression profile was very similar between any *Kit*^*W*^ genotype and *Kit*^+*/*+^ wild-type control; however, in E12.5, a distinct pattern was observed, especially in datasets from *Kit*^*W/W*^ and *Kit*^+*/*+^ (Fig. 2a). In E12.5, 3088 genes (FDR < 0.05) were significantly differentially expressed in *Kit*^*W/W*^ brains compared to *Kit*^+*/*+^ wild-type brains, whereas only 105 genes were significantly differentially expressed in *Kit*^*W/*+^ brains, including 93 genes common with *Kit*^*W/W*^ (Fig. 2b). Four of the five downregulated genes in E15.5 *Kit*^*W/W*^ brains are genes expressed in immature erythrocytes that coincide well with the anemic state in *Kit*^*W/W*^ embryos^23^. We also detected no significant differentially expressed genes within the individual brain of E12.5 *Kit*^*W/W*^ embryos (data not shown), indicating that the diverse gene expression changes observed in the E12.5 *Kit*^*W/W*^ brain occurred uniformly in a developmental stage-specific manner in every E12.5 *Kit*^*W/W*^ embryos.

**Figure 3:**
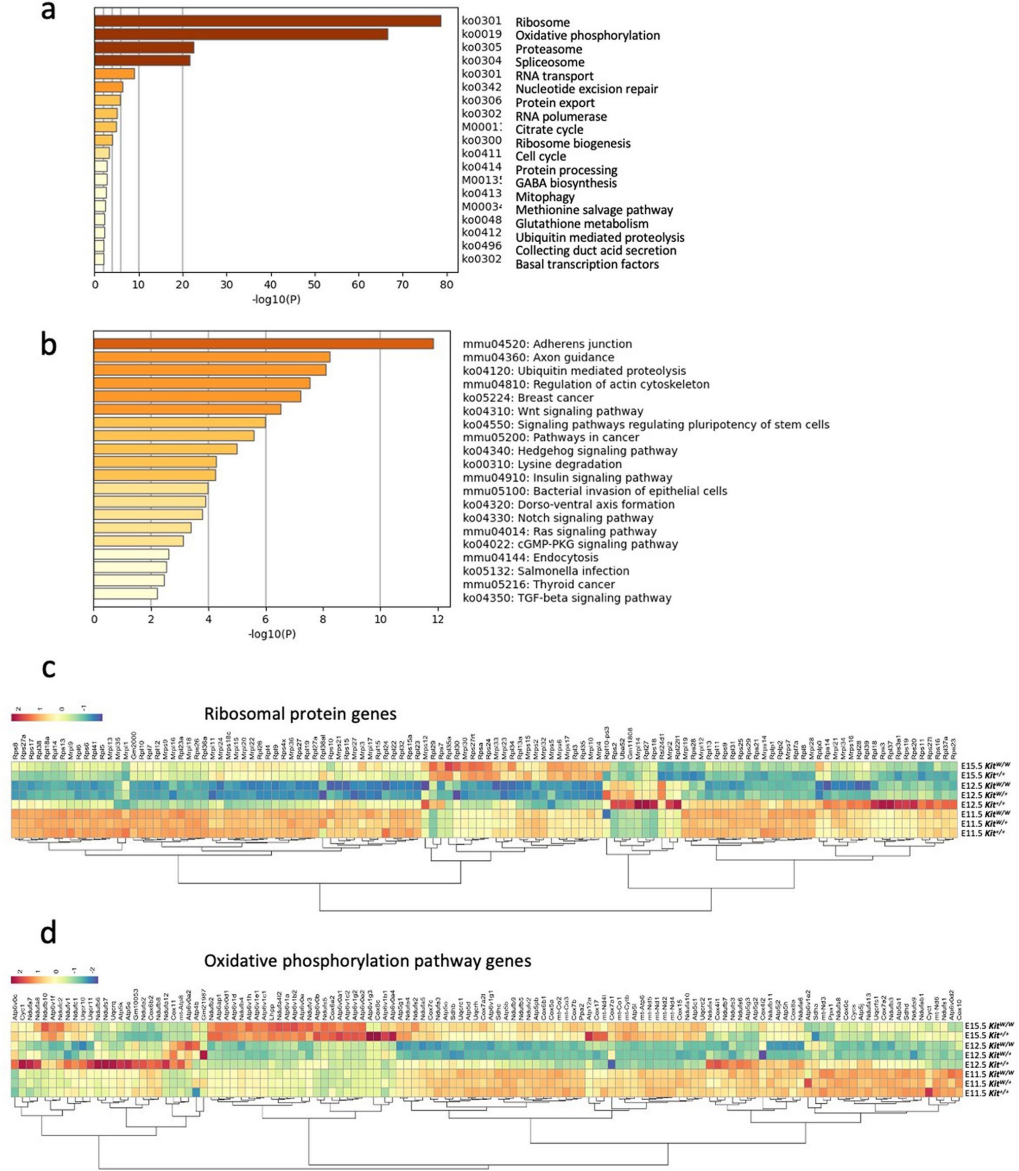
Transcriptomic analysis reveals *Kit*^*W/W*^ brain specific prominent downregulation of genes encoding ribosomal protein and components of the oxidative phosphorylation pathway. (**a**). The 20 top-scoring clusters of significantly enriched (*p* < 0.05) GO terms (KEGG pathway, category: biological process) associated with the downregulated genes in E12.5 *Kit*^*W/W*^ brains compared to E12.5 *W*^+*/*+^ control from significantly DEGs indicated in Fig. 2a. (**b**). Significantly enriched GO terms associated with upregulated genes analyzed as in (**a**). (**c**). Heat map showing the relative expression levels of 120 ribosomal protein genes included in significantly DEGs in E12.5 *Kit*^*W/W*^ brains of Fig. 2a. (**d**). Heat map showing the relative expression levels of 126 genes related with oxidative phosphorylation as in **c**.

We then searched for functional clues in the set of genes differentially expressed in the E12.5 *Kit*^*W/W*^ brain. Gene ontology (GO) terms that were extremely enriched (*p* < 10^70^) were ribosome and oxidative phosphorylation associated with genes downregulated in E12.5 *Kit*^*W/W*^ (Fig. 3a). These GO terms were not shared with those enriched in the datasets from E11.5, E12.5, and E15.5 *Kit*^+*/*+^ wild- type brains (Cluster 4 presented in Supplemental Fig. 4), suggesting that they are closely associated with the homozygous *Kit*^*W/W*^ genetic background.

As indicated in the scatter plots in Fig. 2a, ribosomal protein genes that were highly expressed in the developing brain were uniformly downregulated in the E12.5 *Kit*^*W/W*^ brain. Of the 78 nuclear ribosomal protein genes, 68 were most downregulated in *Kit*^*W/W*^ brains, and one of them was most downregulated in *Kit*^*W/*+^ brains (Fig. 3c). The remaining nine nuclear ribosomal protein genes were not differentially expressed between E12 *Kit*^*W/W*^, *Kit*^*W/*+^, and *Kit*^+*/*+^ brains. This tendency was also observed in mitochondrial ribosomal proteins:17 of those in *Kit*^*W/W*^ brains and 10 in *Kit*^*W/*+^, out of the 41 tested, showed their lowest expression in E12.5 brains (Fig. 3c). Considering the top 10 highly expressed ribosomal protein genes in E 11.5 to E15.5 brains, those of E12.5 *Kit*^*W/W*^ brains are significantly downregulated (Supplemental Fig. 5).

Oxidative phosphorylation was the other prominent term (Fig. 3a), and 72 genes were downregulated of a total of 126 genes in the depicted pathway in E12.5 *Kit*^*W/W*^ brains (Fig. 3d). Key enzymes comprising the respiratory chain and oxidative phosphorylation pathway such as NADH dehydrogenases, cytochrome c oxidases, cytochrome c reductases, F-type ATPases are included in these downregulated genes (Supplemental Fig. 6). Other GO terms enriched in downregulated gene datasets from E12.5 *Kit*^*W/W*^ and *Kit*^+*/*+^brains, namely proteasome, spliceosome, RNA transport, nucleotide excision repair, protein export, RNA polymerase, and ribosome biogenesis, are visualized as a heat map in Supplemental Fig. 7.

GO terms in the datasets of the specifically upregulated genes in E12.5 *Kit*^*W/W*^ brains (Fig. 3b) were not as enriched as those of the downregulated genes. In the 63 genes involved in the most enriched term “adherens junction,” only 22 were differentially expressed but not uniformly changed compared to those in GO terms of the downregulated genes (Supplemental Fig. 8).

### Expression of candidate genes expected to directly compensate Kit activity in developing *Kit*^*W*^ mutant brain

**Figure 4:**
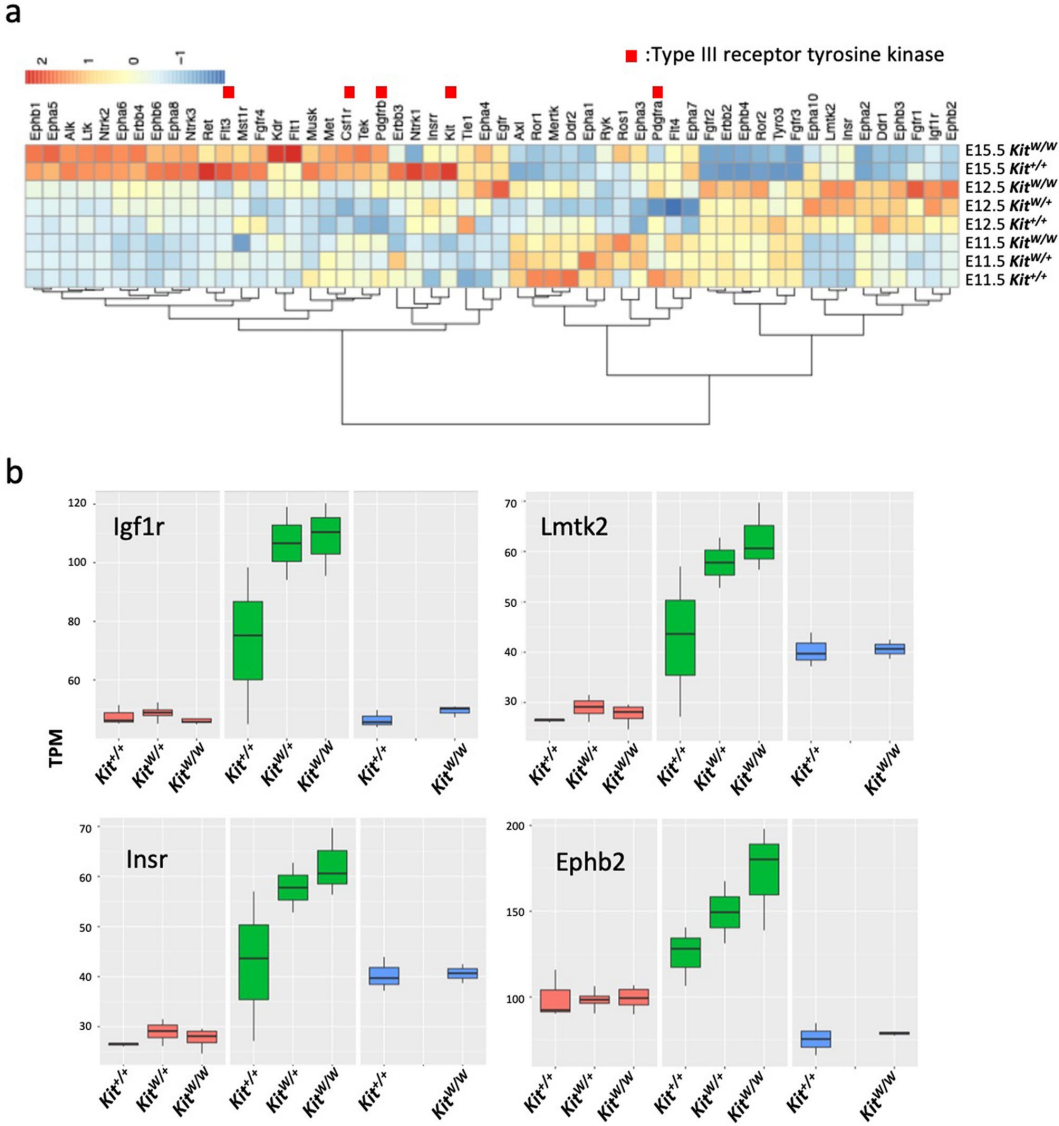
Expression of receptor tyrosine kinase genes homologous with Kit in *Kit*^*W*^ mutant brains. (**a**) Heat map showing the relative expression levels of all 53 Kit paralog genes selected by Ensemble (http://www.ensembl.org/index.html) within all the genes covered by RNAseq. (**b**)**.** Box plot of the significantly upregulated genes (with the median value and 25th and 75th percentiles) in (**a**). Black bar in each box shows the mean of the analyzed gene. TPM: Transcript per million.

It could rationally be anticipated that genes functionally related to *Kit*, in this case most possible candidates are type III receptor tyrosine kinases such as *Csf1R*^24^, could be upregulated in the developing *Kit*^*W*^ brain to compensate for the *Kit*^*W*^ loss-of-function mutation. Elucidated molecular mechanisms, designated as transcriptional adaptation^20^, might explain the upregulation of adapting type III receptor tyrosine kinase genes driven by the *Kit*^*W*^ mutant mRNA decay products expected from the abnormal mRNA containing intronic sequences and premature termination codons (Fig. 1b). However, distinct GO terms related to receptor tyrosine kinase genes did not appear in the datasets of the upregulated genes in the E12.5 *Kit*^*W/W*^ brain; however, we listed all receptor tyrosine kinase genes irrespective of their expression pattern and prepared a heatmap (Fig. 4a). None of the type III receptor tyrosine kinase genes, including *Kit*, was upregulated in the E12.5 *Kit*^*W/W*^ brain. Four other genes were significantly differentially upregulated (Fig. 4b), but none of them has been reported to be replaceable with *Kit*, at least in part. Interestingly, the key nonsense-mediated decay factor Upf1 and several other related genes necessary for transcriptional adaptation^9,10^ were upregulated specifically only in E12.5 *Kit*^*W/W*^ and *Kit*^*W/*+^ brains (Supplementary Fig. 9).

Signaling modulators of receptor tyrosine kinases, including various cytoplasmic tyrosine kinases and tyrosine phosphatases, may also compensate for the *Kit*^*W*^ loss-of-function mutation in the developing brain^25^. Of these modulator genes, 36 were differentially expressed specifically in the E12.5 *Kit*^*W/W*^ and *Kit*^*W/*+^ brains, of which 13 were upregulated and the remaining 23 were downregulated (Supplementary Fig. 10).

## Discussion

Two-thirds of mouse genes tested for their phenotypes after induction of homozygous loss-of-function mutation showed no prominent phenotype and mice lived in adulthood^1^. This apparent paradox for the Darwinian gene definition, in which each gene must be susceptible to natural selection and thus indispensable and hard to be deleted has been reconciled by the presence of a functionally redundant set of genes and their possible stability against strong selection pressure for generations, at least in several specific conditions^3–7^. Although *Kit* and its ligand (*Kitl*) are highly expressed in the brain, the lack of major defects in the structure and function of the central nervous system in various *Kit* homozygous loss-of-function mutants has long been considered a typical case of genetic redundancy^17,18^.

Recently, “transcriptional adaptation” has been proposed as the molecular mechanism supporting this genetic redundancy or robustness theory^20^. In this process, decayed mutant mRNA fragments derived from the prematurely terminated mutant gene guide a specific protein complex to the homologous part of the compensatory gene to enhance its transcription. In the case of the *Kit*^*W*^ mutation, splicing defects induced by the point mutation at the splice acceptor site produce a nascent termination codon in the readthrough of intron10 in mRNA (Fig. 1b), which may induce nonsense-mediated mRNA decay of the premature *Kit* primary transcript^26^.

However, as described in the Results section, type III receptor tyrosine kinase genes that are most homologous to *Kit* were not transcriptionally augmented. Other receptor tyrosine kinases, such as Igf1r, Lmtk2, Insr, and Ephb2, were significantly upregulated in E12.5 *Kit*^*W/W*^ brains. Igf1r, Lmtk2, and Insr were significantly upregulated, even in E12.5 heterozygous *Kit*^*W/*+^ brains (Fig. 4), consistent with the dominant nature of transcriptional adaptation^9^. Injection of mouse mRNA induces transcriptional adaptation even for homologous zebrafish genes, as the sequence similarity required to induce transcriptional adaptation is not so stringent; therefore, the above genes could possibly be upregulated by transcriptional adaptation. Nevertheless, the overall structures of the upregulated genes are not related to *Kit*, except for the tyrosine kinase domain, and are not likely to directly compensate for *Kit* function. Furthermore, these genes were only upregulated in E12.5 *Kit*^*W/W*^ brains but not in E11.5 and E15.5 *Kit*^*W/W*^ brains. In the reported transcriptional adaptation reaction, compensatory gene expression continues much longer, at least from 22 to 78 h after fertilization in zebrafish, or even after the injection of responsible RNA fragments in various stages of the embryo and cultured cell line^9^. Therefore, the observed developmental stage-restricted upregulation of these genes in the *Kit*^*W*^ mutant does not represent a typical transcriptional adaptation reaction, since defective *Kit* mRNA is synthesized throughout life in the brain. Notably, some genes related to mRNA decay were upregulated specifically in E12.5 *Kit*^*W/W*^ brains (Supplemental Fig. 9), suggesting that the transcriptional adaptation reaction itself could be enhanced in E12.5.

Signaling modulators of receptor tyrosine kinase may well compensate for the *Kit*^*W*^ mutation, as reported regarding *Kit* gain-of-function mutation-induced tyrosine phosphatase SHP-1 degradation^27^. However, at least in the *Kit*^*W/W*^ mutant, no functional Kit receptors are produced, and therefore, any modulations to enhance the net activity of Kit tyrosine kinase activity may be ineffective. Still, feedback activation of the other synergistically acting signalings such as M-CSF, GM-CSF, IL-3 may compensate for the lack of Kit signal in *Kit*^*W/W*^ brain.

The GO terms most enriched in the differentially expressed genes in the E12.5 *Kit*^*W/W*^ brains were ribosomal proteins. Most of the nuclear ribosomal proteins (63 out of 78) were included in the 1632 significantly downregulated genes in E12.5 *Kit*^*W/W*^ brains in comparison with E12.5 *Kit*^+*/*+^ control brains. Downregulation was most prominent in E12.5 *Kit*^*W/W*^ homozygous brains and tended to be observed in E12.5 *Kit*^*W/*+^ heterozygous brains (Supplemental Fig. 5). Owing to the loss of growth factor signaling such as *Kit*, apoptotic processes could be activated in neural cells as in hematopoietic cells^28,29^. Apoptotic death of neuronal cells was detected in the brain soon after the induced haploinsufficiency of the *Kit*^13^. If genetic compensation by functionally redundant gene(s) does not occur, as discussed above, other mechanisms circumventing this fatal situation through cell cycle arrest and reduction of cell growth and differentiation^30^ are expected to take place in E12.5 *Kit*^*W/W*^ brains. This type of protection from apoptosis is achieved by protein synthesis inhibitors^31–33^. Slowing down metabolic processes, including protein synthesis, is a strategy used to extend the dormant state of stem cell populations to avoid apoptosis, although in a highly controlled manner^34,35^. In neural stem cells, the expression of ribosome biosynthesis-related genes is lowest in their dormant state and quickly increases upon injury-induced activation^36^. Transient repression of transcriptional activity has been observed in primordial germ cells of sea urchin embryos^37^. The direct relationship between protein synthesis and hematopoietic cells at various stages has been investigated in vivo, and the hematopoietic stem cell population showed the lowest protein synthesis rate per hour than in most other differentiated hematopoietic cells^38^. In addition, the increased rate of protein synthesis observed in hematopoietic stem cells of *Pten* knockout mice was restored to normal levels by introducing a loss of function mutation in the ribosomal protein *Rpl24* and leukemogenesis accompanying *Pten* deficiency was suppressed at the same time^39^, indicating a direct relationship between the levels of ribosomal proteins and protein synthesis rate and the resultant control of cellular proliferation. Our assumed dependency of brain development on *Kit* signaling around the E12.5 brain and thereby the induction of a catastrophic state in the E12.5 *Kit*^*W/W*^ brain, might be rescued by the reduction of protein synthesis by the observed downregulation of ribosomal proteins.

Oxidative phosphorylation was another prominent GO term enriched among the differentially expressed genes in E12.5 *Kit*^*W/W*^ brains. Most of the constituent genes were downregulated specifically in E12.5 *Kit*^*W/W*^ brains, similar to the ribosomal protein genes. Mitochondrial aerobic metabolism is known to restrict differentiation of hematopoietic stem cells^39^. It is likely that the above-mentioned protective metabolic slowdown caused by the downregulation of ribosomal proteins is further strengthened by the suppression of oxidative phosphorylation attained by the downregulation of related genes. It should be noted that uniform downregulation of genes encoding ribosomal and oxidative phosphorylation pathway proteins was observed not only in the E12.5 *Kit*^*W/W*^ homozygous brain, but also in the E12.5 *Kit*^*W/*+^ heterozygous brain, although this was not statistically significant (Fig. 3d and Supplemental Fig. 5). The fatal effect of *Kit*^*W*^ mutation occurs even in the heterozygous state and seems to be prevented in the germline E12.5 *Kit*^*W/*+^ brain. We previously reported that induced haploinsufficiency in embryonic brains is sufficient to induce severe brain hypoplasia^13^. In this case, sudden deletion of *Kit* gene during embryogenesis might not be fully rescued even by our suggested global gene expression change.

The molecular mechanism responsible for the global reduction in ribosomal protein transcription is currently difficult to address in relation to the loss of Kit signaling in *Kit*^*W/W*^ mutant brains. As we did not detect a significant reduction in ribosome biogenesis gene expression by the pathway analysis between E12.5 *Kit*^*W/W*^ and wild-type brain (18 are downregulated in 65 ribosome biogenesis: Supplemental Fig. 7), the transcription of ribosomal protein genes is supposed to be suppressed. Together with the downregulation of genes related to oxidative phosphorylation, proteasome, and spliceosome (Fig. 3a), uniform compensatory downregulation of ribosomal protein genes may cause reduction of protein synthesis to the minimal rate to circumvent fatal cell death in the E12.5 brain in *Kit*^*W/W*^ germline mutants and maintain neural stem cells in a dormant state until the absolute dependency on Kit signaling is cancelled, as reported in the case of melanocyte stem cells^40^. At least in E15.5, the compensatory gene expression change returned to normal in *Kit *^*W/W*^ brain.

In yeast, the coordinated regulation of ribosomal protein gene transcription is attained by the conserved promoter of each ribosomal protein gene with a restricted number of regulatory proteins^41,42^. We found no significant changes in the mRNA levels of several transcription factors possibly related to ribosomal protein expression (Supplementary Fig. 11a). In ribosomal defects, known collectively as ribosomopathies, induction of p53 activation and expression of the target p21Cip1 (Cdkn1a) have been observed^43^. While the observed downregulation of ribosomal proteins is obviously not a specific functional defect of each ribosomal protein gene, concomitant reduction of the translation activity may cause cell cycle arrest through the induction of p21Cip1 expression in E12.5 *Kit*^*W/W*^ brains; however, we did not observe any difference in p21Cip1 expression compared to E12.5 wild type brains (Supplementary Fig. 11b).

Here, we suggest a mechanism to reconcile the lack of Kit signaling during embryonic growth and differentiation of Kit-dependent neural cells in the brain. The presence of this kind of genetic compensation system or genetic robustness may reduce the fatal effects of mutations in essential genes, such as *Kit*, to support the normal development of a specific organ^13^. From another point of view, owing to this genetic compensation system, a number of *Kit* mutations spreading in vertebrates are maintained in each species. In turn, possible evolutionarily favorable coat colors have been brought by impaired melanocyte development by these *Kit* mutations^44^. As reported previously in mouse melanocytes^40,45,46^, the strict requirement of Kit signaling for cell survival is restricted to a short period of time during the entire developmental period. In the case of *Kit*^*W/W*^ brains, lack of Kit signaling could only be fatal around E12, and the suggested genetic compensatory mechanism functions efficiently only at this stage.

Noticeable phenotypes of many *Kit* mutations are known to be restricted to those that appear in hematopoietic, neural crest, and germ cells; however, recent genome-wide analysis revealed significant levels of *Kit* expression in most cell lineages in mice (http://www.informatics.jax.org/marker/MGI:96677) and humans (https://www.genecards.org/cgi-bin/carddisp.pl?gene=KIT). It is possible that in many other cell lineages expressing Kit, the observed compensatory system works at a certain stage of their development. Considering the fact that almost one-third of the genes are not indispensable, at least as judged by the effects of their homozygous loss-of-function mutations^1^, it is conceivable that our suggested system observed in *Kit*^*W/W*^ mutant is not just a unique case but works in other genes.

We did not mention the top ten DEGs shown in Fig. 2a. For example, *Ints7* is upregulated significantly and specifically in both *Kit*^*W/W*^ and *Kit*^*W/*+^ brains from E12.5. This gene mediates 3'-end processing of small nuclear RNAs. As for the remaining nine genes, there is no evidence or speculation regarding their involvement in the observed genetic compensation of the *Kit*^*W*^ mutation. To further know the prevalence of our finding, differential gene expression in the brain of the other type III receptor tyrosine kinase mutant might be informative. Single cells RNAseq analysis of E12.5 *Kit*^*W/W*^ brain may also be helpful to look at very early phase of the gene compensation process.

## Materials and methods

### Mice

*Kit*^*W/*+^ mice were obtained from Japan SLC (Shizuoka, Japan) and housed in standard animal rooms with food and water ad libitum under controlled humidity (50 ± 10%) and temperature (22 ± 2 °C) conditions. The room was illuminated by fluorescent lights from 8:00 AM to 8:00 PM. For embryo timing, vaginal plugs were checked, and the day a plug was detected was considered as E0.5. All animal experiments were conducted in accordance with the Guide for the Care and Use of Laboratory animals approved by the Animal Care Committee of the Gifu University which followed the ARRIVE guidelines.

### Preparation and sequencing of cDNA

Total RNA was prepared from the whole brain of each embryo by using the RNeasy Plus Mini Kit (Qiagen), according to the manufacturer’s instructions. RNA-seq library preparation and DNBseq platform sequencing of the extracted total RNA were performed by the BGI Group (Shenzhen, China).

### Read data processing and alignment

Sequenced read data were filtered, trimmed, error-removed, and quality checked using AfterQC (version 0.9.6)^47^. The filtered reads were mapped to the genome of Mus musculus (Mus_musculus.GRCm38.dna_sm.primary_assembly.fa derived from Ensembl) using HISAT2 (version 2.1.0)^48^. The alignment results were visualized using the Integrative Genomics Viewer (IGV). The SMA files produced by mapping were sorted and converted into BAM files using SAMtools. The number of reads mapped to each gene was counted based on the gene annotation (Mus_musculus.GRCm38.100.chr.gtf.gz) corresponding to the reference genome using featureCounts included in the Subread package (version 2.0.3) with the following parameters: “-p –countReadPairs -B -C”. A matrix of raw counts per gene for each sample was prepared for the gene expression analysis.

### Identification of differentially expressed genes

The significantly differentially expressed genes (DEGs) were identified using the R-based package EdgeR (version 3.28.0)^49^. Raw count data were filtered using the FilterByExpr function and normalized using the calcNormFactors function. The DEGs were identified from the pairwise comparisons of *Kit*^+*/*+^ versus *Kit*^*W/W*^ and *Kit*^*W/*+^ in E11.5, E12.5, and E15.5, using quasi-likelihood F-tests with a false discovery rate (FDR) < 0.05. Other DEGs were extracted from the ANOVA-like comparison among E11.5, E12.5, and E15.5 *Kit*^+*/*+^ using quasi-likelihood F-tests with FDR < 0.05.

### Gene expression data exploration and visualization

For each gene, the expression levels in each sample were calculated as transcripts per million (TPM). For each filtered-out gene, the base 10 logarithm of the mean TPM for the same condition after adding 1 was calculated, and the distribution was summarized in a box plot. Similarities across conditions based on these values were explored using PCA and visualized in a scatter plot. For each filtered-out gene, the base 2 logarithm of the mean TPM for the same condition after adding 1 was calculated. The ratio and mean of these values for each gene were plotted as an MA plot for each pairwise comparison. The TPMs for individual genes under each condition are summarized in box plots. Z-scores were calculated for each gene and hierarchical clustering was performed based on the expression pattern of each gene, which was visualized in a heatmap, dendrogram, and trend line. Except for the heatmap generated by the pheatmap function included in the R package, all others were drawn using the R package ggplot. Gene set enrichment analysis (GSEA) was performed using Metascape^50^ in the Kyoto Encyclopedia of Genes and Genomes (KEGG) database^51–53^. Z-scored gene expression was plotted on a pathway map derived from KEGG using Pathview Web^54^.

### Study approval

All animal experiments were approved by the Animal Care Committee of the Gifu University, Gifu, Japan (approval number: 24-19 and 26-44).

## Supplementary Information

Supplementary Figures.

## Data Availability

The sequencing read data analyzed in this study were deposited in the DDBJ Sequencing Read Archive (DRA) under the accession number DRA014628.
