# Supplementary material for: Compensatory gene expression potentially rescues impaired brain development in Kit mutant mice
Source: Sci Rep. 2023 Mar 13;13:4166. doi: 10.1038/s41598-023-30032-0 (PMC10011532; doi:10.1038/s41598-023-30032-0)

Supplementary Information

**Legends for supplementary figures**

**Supplemental Figure 1. Morphology and neural cell properties of mice induced brain-specific Kit haploinsufficiency. a.** Embryos were sectioned around the indicated lines. In Sox1-Cre; Kit^2lox/+^ embryos, most areas of the brain were degenerated compared with their normal counterparts. **b.** Cells harvested from the brain and spinal cord of E12.5 *Sox1-Cre; Kit^2lox/+^; Rosa26R- EYFP* embryos and control *Sox1-Cre; Kit^+/+^; Rosa26R-EYFP* embryos were analyzed using flow cytometry. The hematopoietic cell marker CD45 and a Sox1-Cre- induced neural cell lineage tracer, detected as EYFP, were used to identify neural cells. Cell surface kit expression was measured in CD45-negative and EYFP-positive cells.

**Supplemental Figure 2. Principal component analysis (PCA) of brain cells of the indicated *Kit^W^* mutant mice based on RNAseq analysis shown in Figure 2a.** Brain cells were clustered according to their developmental stages, irrespective of their genotypes.

**Supplemental Figure 3. Sequence read histograms of individual mouse embryos used for RNA-seq analysis.** Each histogram pattern of the embryo corresponds well with the genotype determined independently by RT-PCR, as indicated on the right of each histogram.

**Supplemental Figure 4. Developmental changes in gene expression in *Kit^+/+^* wild type brains. a.** Expression dynamics of 14218 DEGs within wild-type mouse brains by RNAseq (FDR<0.01 by ANOVA one-way test) categorized as clusters 1 to 5 according to the stage-specific expression pattern. **b.** The 32 top-scoring clusters of significantly enriched (p<0.05) GO terms (KEGG pathway, category: biological process) were associated with cluster 4 gene set, which was specifically downregulated in E12.5, similar to the ribosomal protein genes in the E12.5 *Kit^W/W^* brain.

**Supplemental Figure 5. Quantification of ribosomal protein gene expression.** Box plot of the 10 significantly downregulated ribosomal protein genes (with the median value and the 25th and 75th percentiles) shown in Fig.3c. The black bar in each box shows the mean of the analyzed genes. TPM: transcripts per million.

**Supplemental Figure 6. The expression of 126 genes regulating oxidative phosphorylation included in the significantly DEGs in E12.5 *Kit^W/W^* brains were compared with those of *Kit^W/+^* and *Kit^+/+^*. E**ach gene was named within the box and separated into three columns representing the status of *Kit^+/+^, Kit^W/+^, and Kit^W/W^* brains at E12.5. Most genes showed the lowest expression in the *Kit^W/W^* brain. Some genes are indicated in the picturelized oxidative phosphorylation pathway.

**Supplementary Figure 7. Expression of genes within GO terms enriched in downregulated datasets from E12.5 *Kit ^W/W^* and *Kit^+/+^*brains.** Heat map showing the relative expression levels of the indicated genes consisting GO terms in Figure 3a except ribosomal protein and oxidative phosphorylation are shown.

**Supplementary Figure 8. Expression of genes within most enriched GO term “cell adhesion” in upregulated datasets from E12.5 *Kit ^W/W^* and *Kit^+/+^*brains.** Heat map showing the relative expression levels of the indicated genes.

**Supplemental Figure 9. Quantification of genes related to nonsense-mediated decay factors.** Box plot of the three significantly upregulated genes (with the median value and 25th and 75th percentiles) related to non-sense-mediated decay. The black bar in each box shows the mean of the analyzed genes. TPM: transcripts per million.

**Supplemental Figure 10. Expression of receptor tyrosine kinase signaling modulators in *Kit^W^* mutant brains. a.** Heat map showing the relative expression levels of modulators of receptor tyrosine kinase within all the genes covered by RNAseq. **b.** Box plot of the significantly upregulated genes (with the median value and 25^th^ and 75^th^ percentiles) in **a** black bar in each box shows the mean of the analyzed genes. TPM: transcripts per million.

**Supplemental Figure 11. Quantification of transcriptional regulation genes related to ribosomal protein expression. a.** Box plot of five transcriptional regulation genes possibly related to ribosomal proteins (with the median value and 25th and 75th percentiles). **b**. Quantification of Cdkn1a gene possibly related to ribosomal protein expression. The black bar in each box shows the mean of the analyzed genes. TPM: transcripts per million.


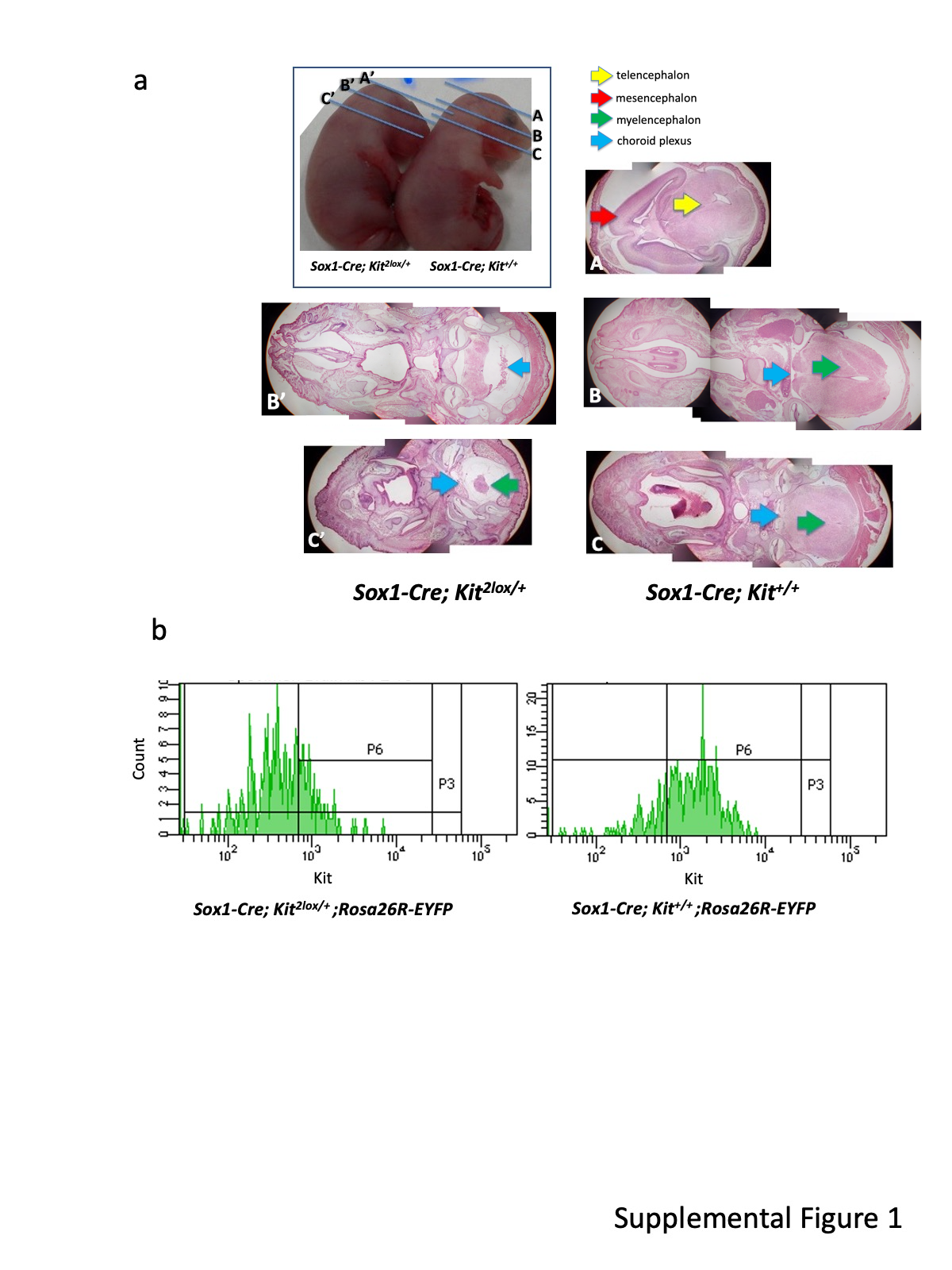


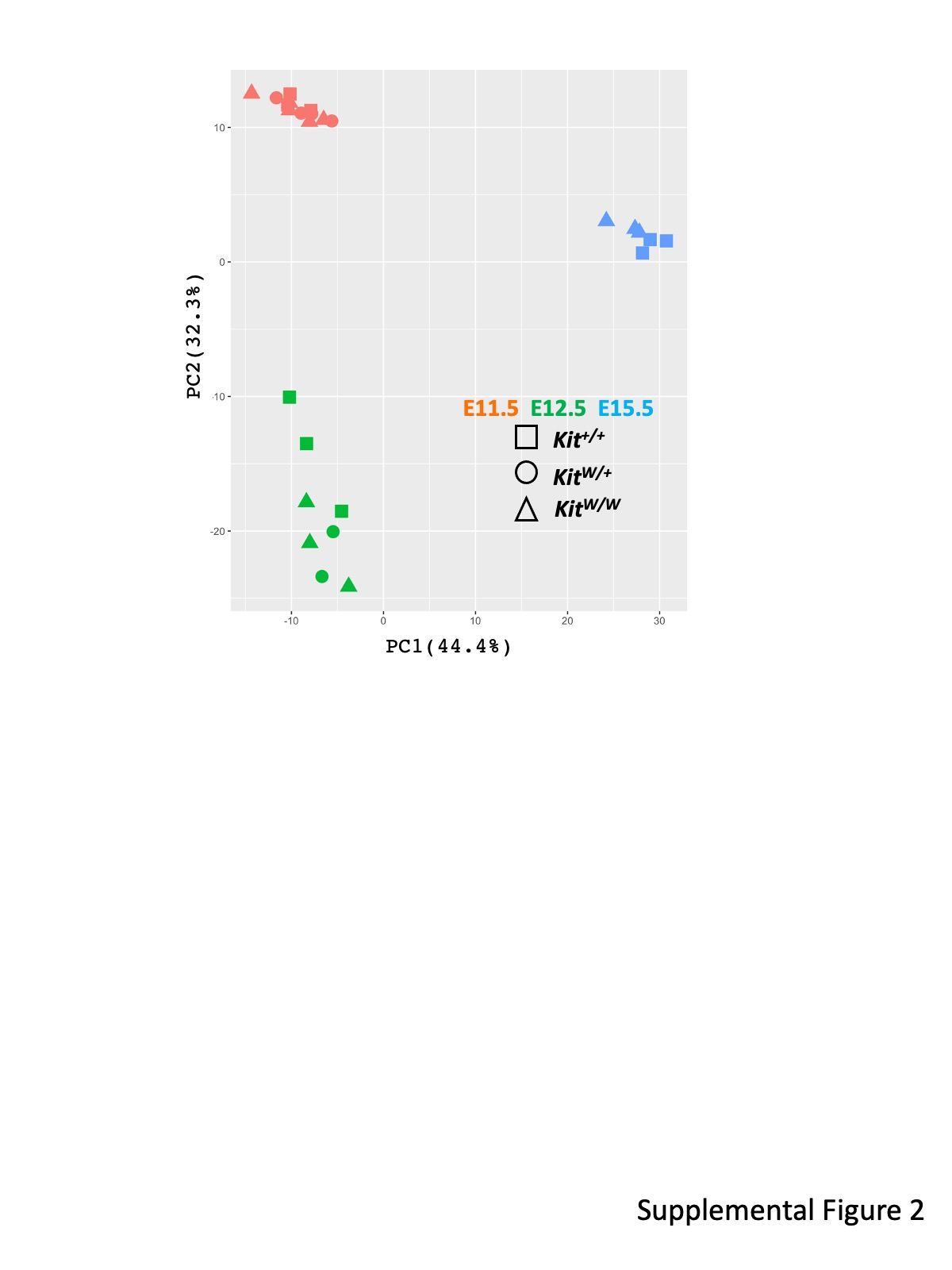


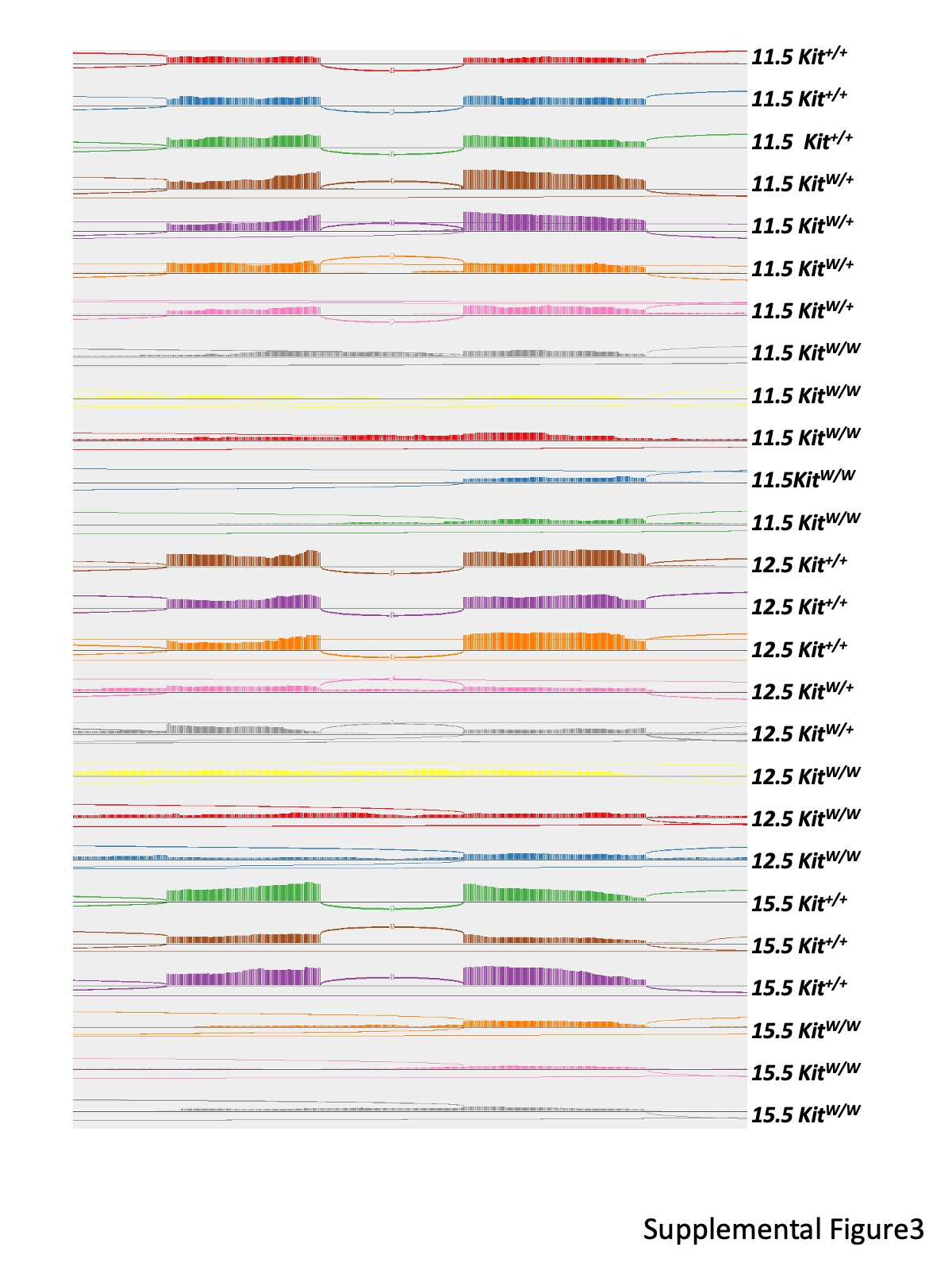


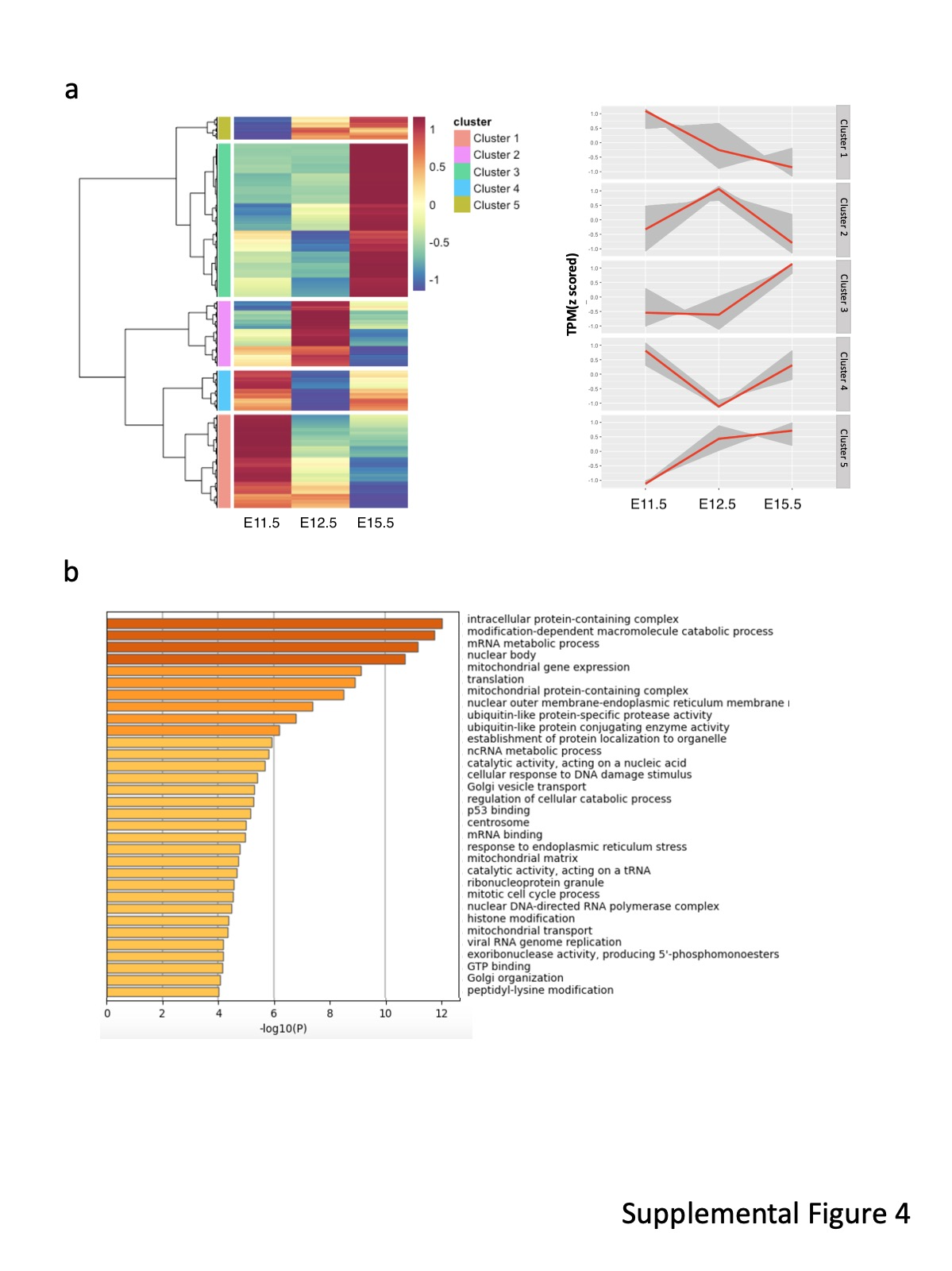


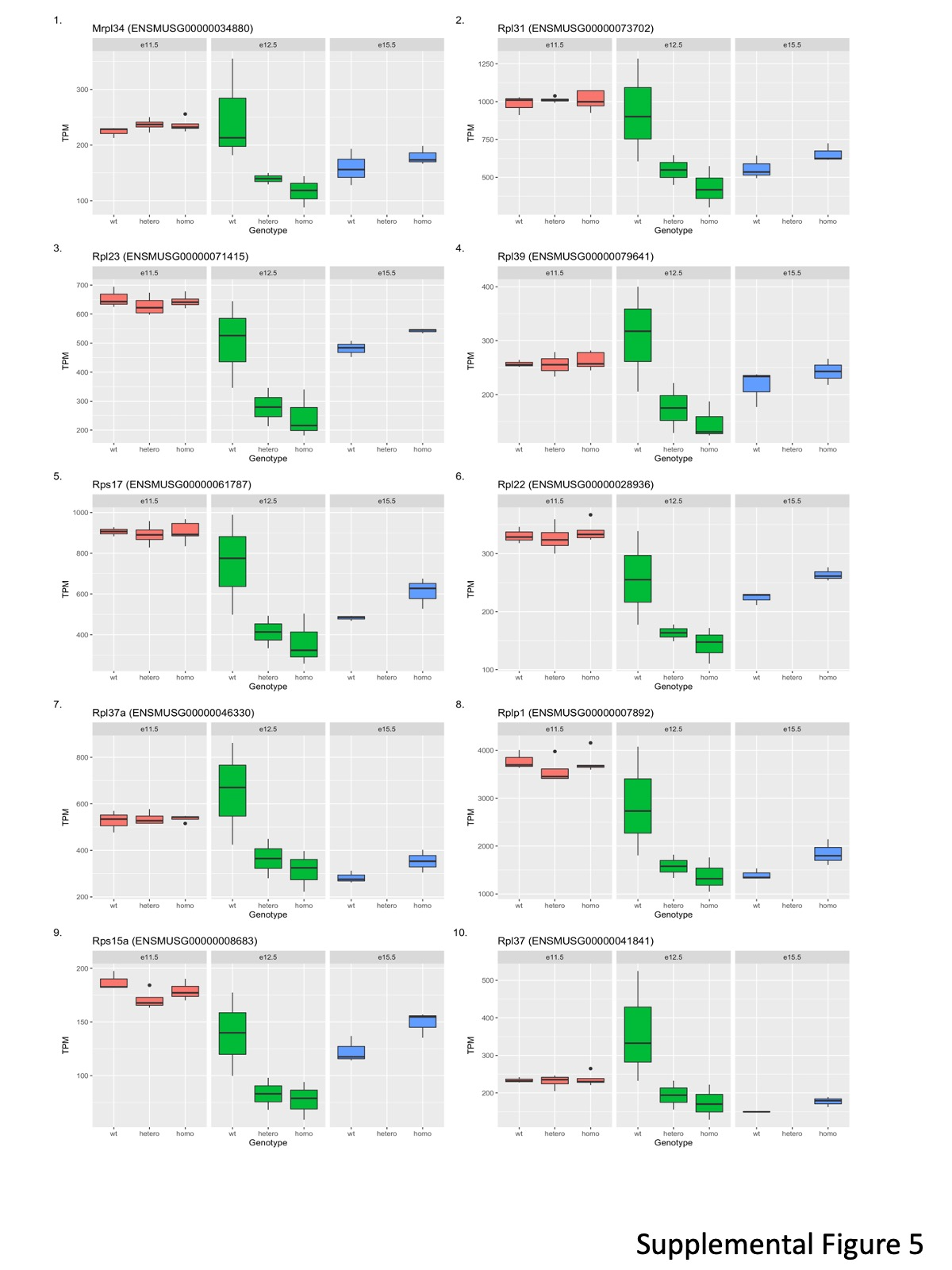


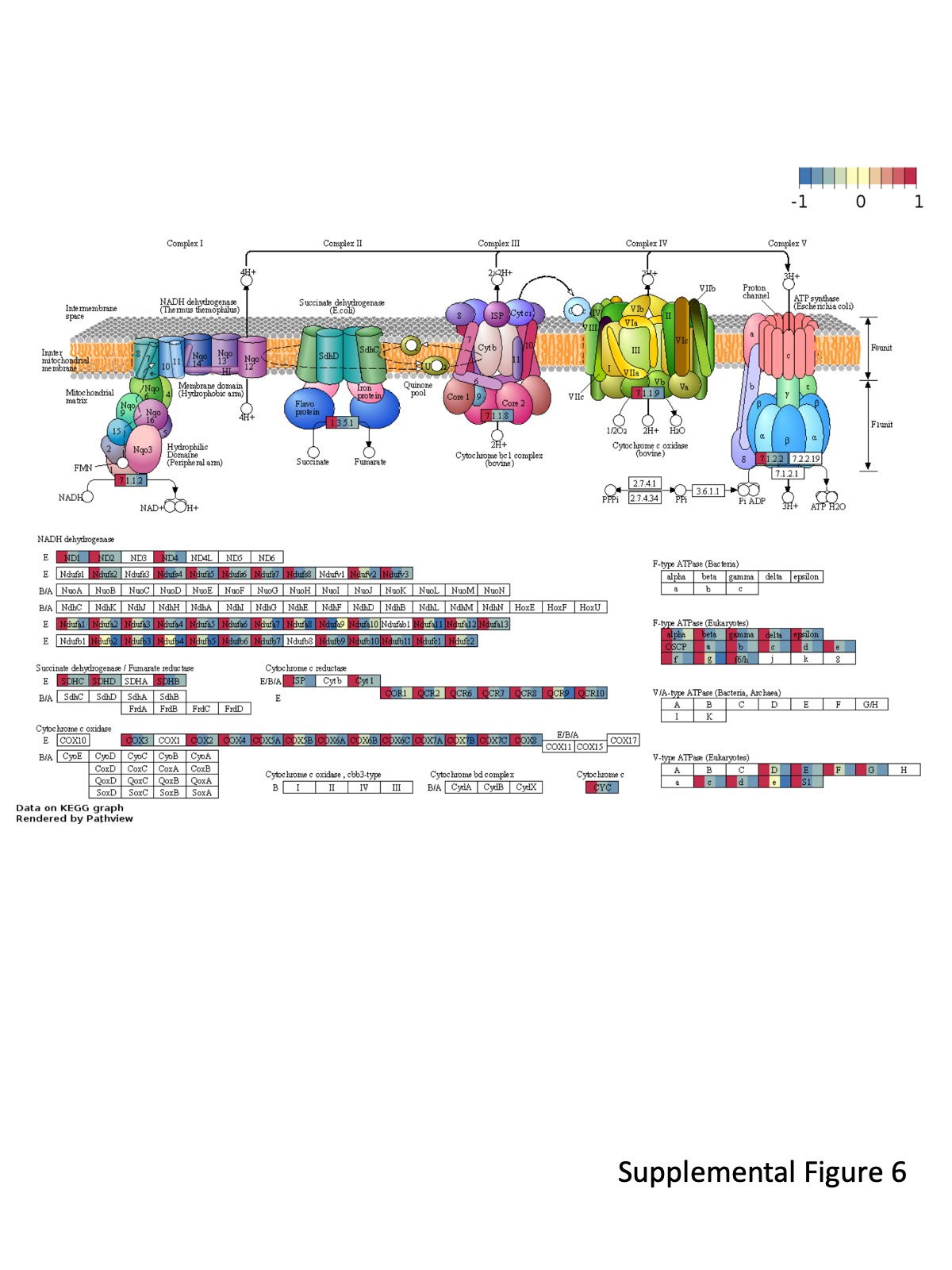


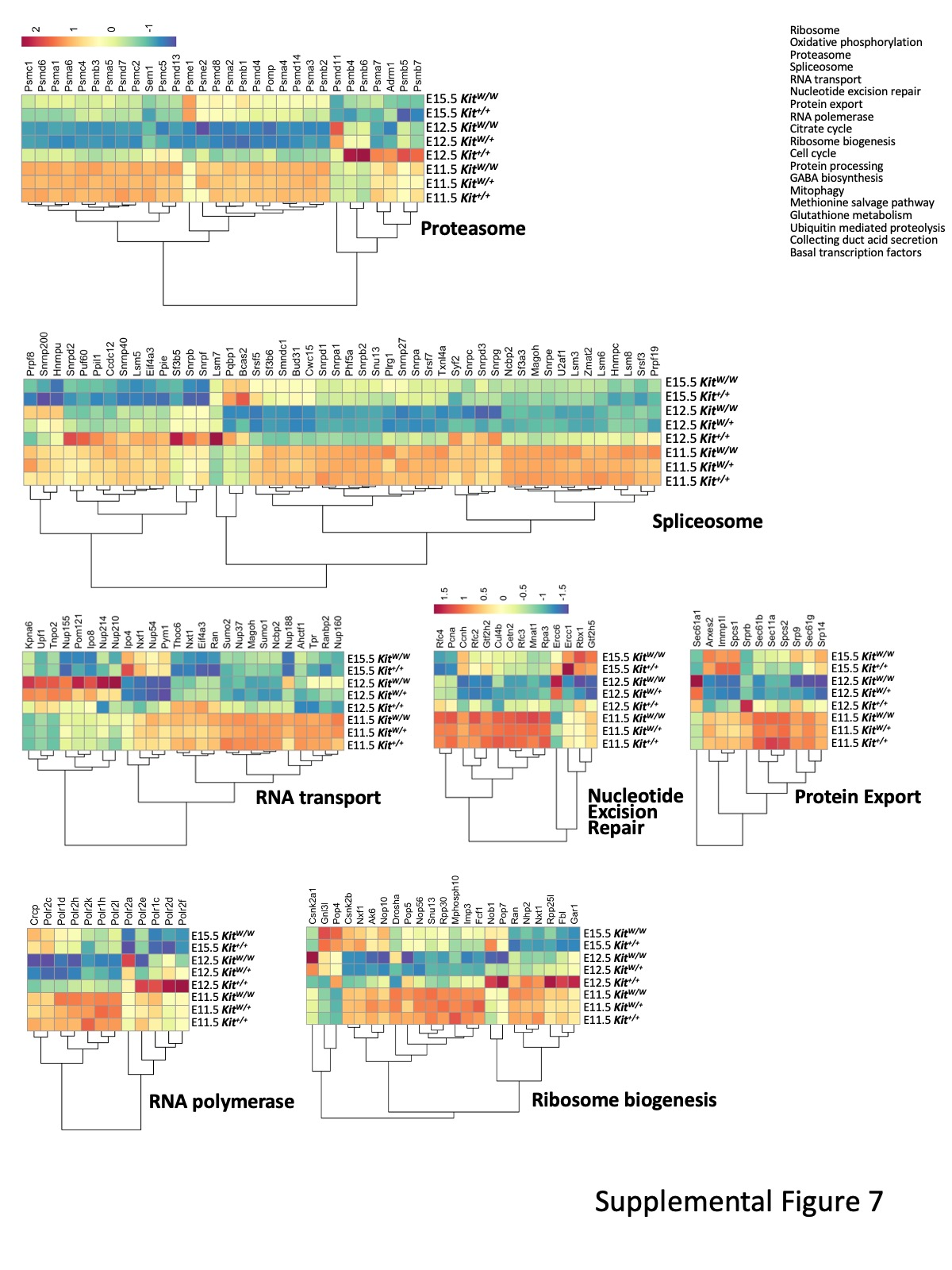


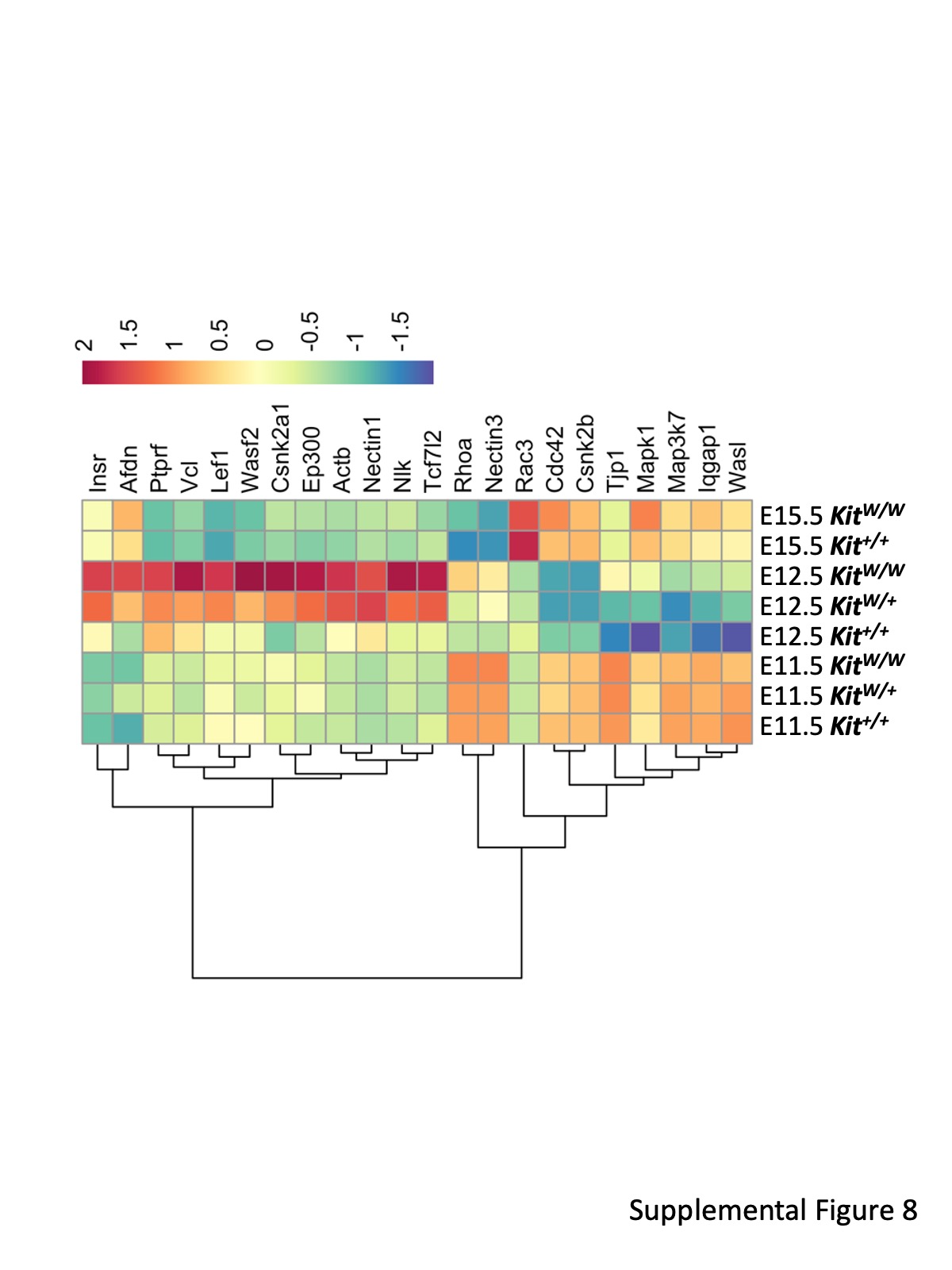


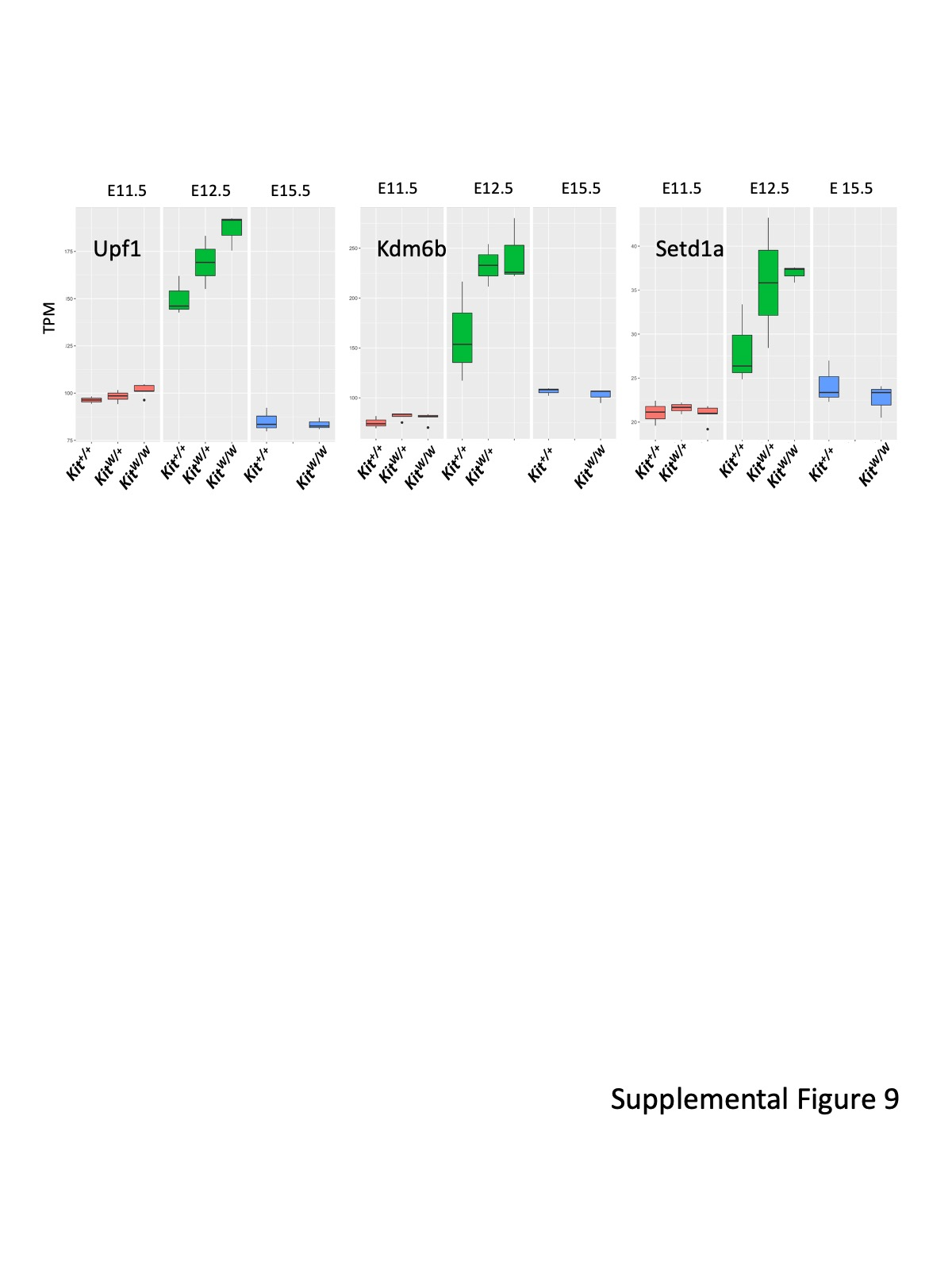


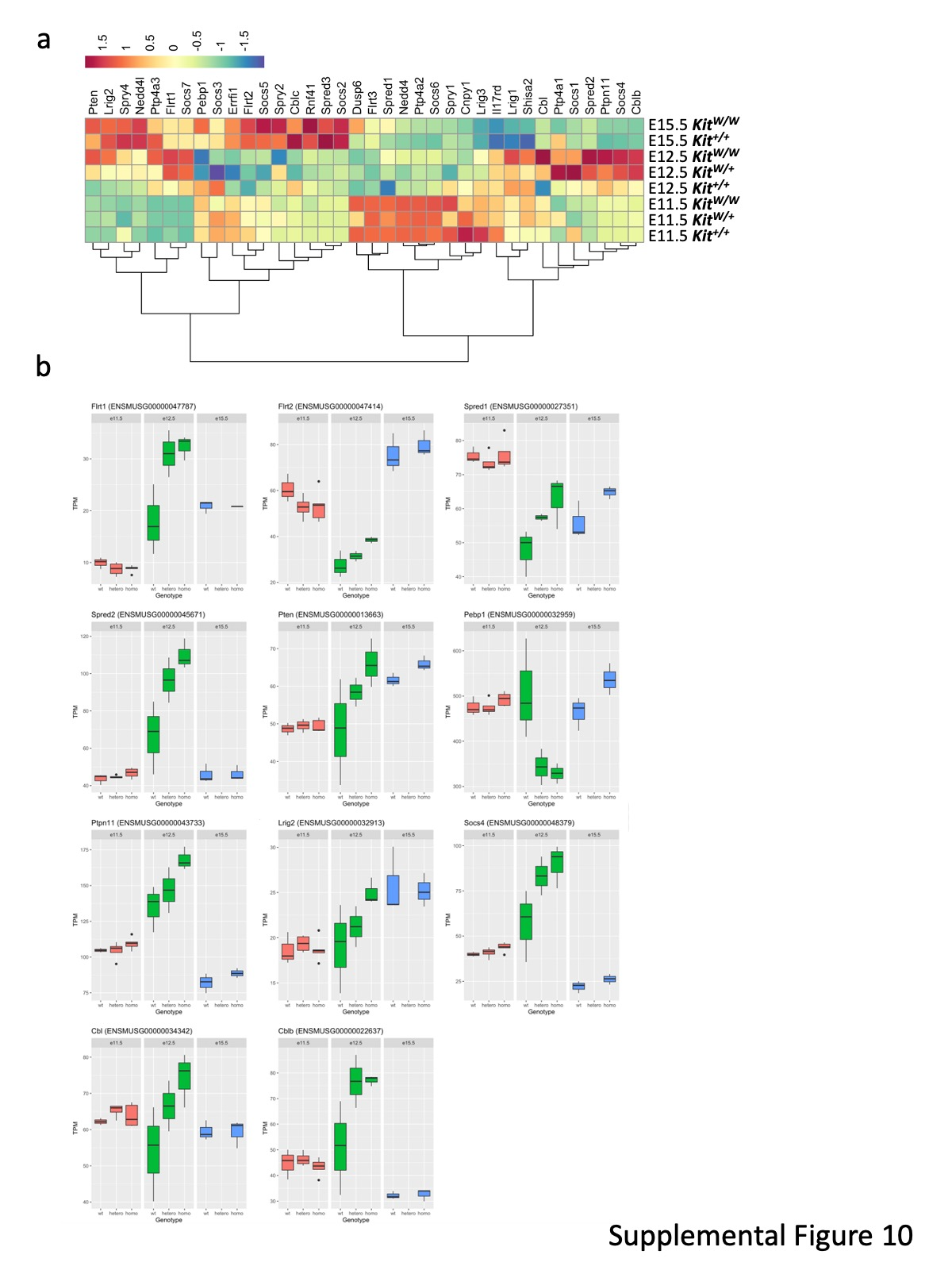


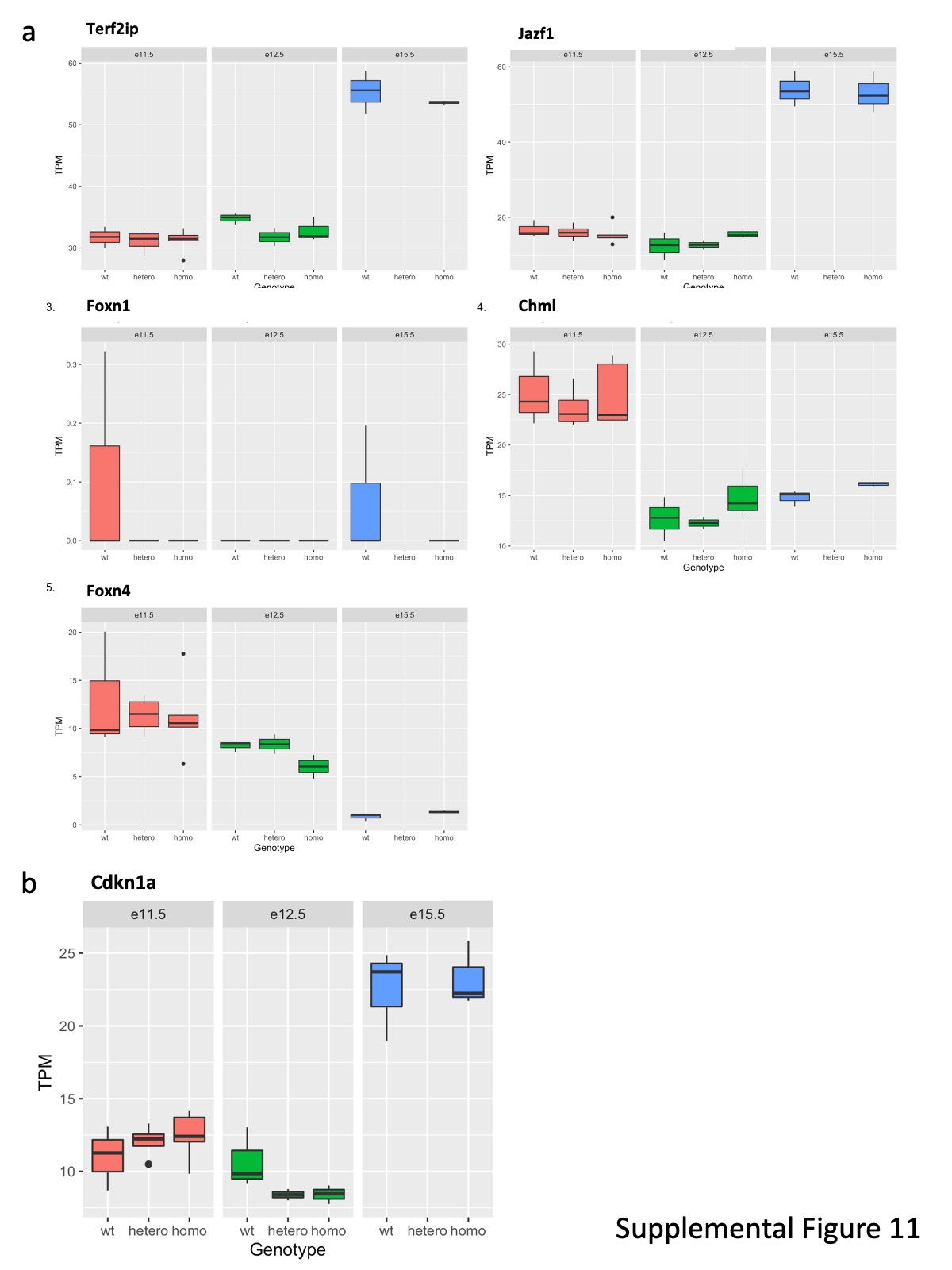

Supplement: Supplementary file 1 — Supplementary Figures. [file 41598_2023_30032_MOESM1_ESM.docx]
